# Estimating the Product of the X-ray Spectrum and Quantum Detection Efficiency of a CT System and Its Application to Beam Hardening Correction

**DOI:** 10.3390/s21093284

**Published:** 2021-05-10

**Authors:** Joseph J. Lifton, Andrew A. Malcolm

**Affiliations:** Advanced Remanufacturing and Technology Centre, 3 Cleantech Loop, CleanTech Two, Singapore 637143, Singapore; andrew_malcolm@artc.a-star.edu.sg

**Keywords:** X-ray spectrum, quantum detection efficiency, beam hardening, Bézier curve, linearisation

## Abstract

Lab-based X-ray computed tomography (XCT) systems use X-ray sources that emit a polychromatic X-ray spectrum and detectors that do not detect all X-ray photons with the same efficiency. A consequence of using a polychromatic X-ray source is that beam hardening artefacts may be present in the reconstructed data, and the presence of such artefacts can degrade XCT image quality and affect quantitative analysis. If the product of the X-ray spectrum and the quantum detection efficiency (QDE) of the detector are known, alongside the material of the scanned object, then beam hardening artefacts can be corrected algorithmically. In this work, a method for estimating the product of the X-ray spectrum and the detector’s QDE is offered. The method approximates the product of the X-ray spectrum and the QDE as a Bézier curve, which requires only eight fitting parameters to be estimated. It is shown experimentally and through simulation that Bézier curves can be used to accurately simulate polychromatic attenuation and hence be used to correct beam hardening artefacts. The proposed method is tested using measured attenuation data and then used to calculate a beam hardening correction for an aluminium workpiece; the beam hardening correction leads to an increase in the contrast-to-noise ratio of the XCT data by 41% and the removal of cupping artefacts. Deriving beam hardening corrections in this manner is more versatile than using conventional material-specific step wedges.

## 1. Introduction

**Figure 1:**
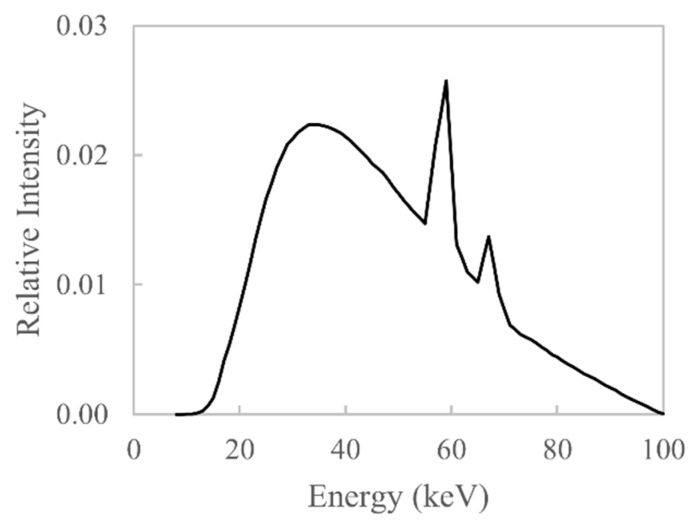
A simulated polychromatic X-ray spectrum emitted by a tungsten reflection target X-ray source, obtained using the model in [7].

The X-ray sources used in lab-based X-ray computed tomography (XCT) systems generate a polychromatic X-ray spectrum such as that shown in Figure 1. Furthermore, the indirect flat-panel X-ray detectors used in lab-based XCT systems do not detect all X-ray photon energies with the same efficiency. As a consequence of using these types of X-ray sources, a non-linear relationship between X-ray attenuation and material thickness is found. This non-linear relationship stems from the fact that X-rays of different energies are attenuated to varying degrees; generally speaking, lower-energy X-rays are more easily attenuated than higher-energy X-rays. If the relationship between X-ray attenuation and material thickness is non-linear, then there will be errors (artefacts) in the reconstructed XCT data. Non-linear X-ray attenuation is particularly severe for materials with high atomic numbers and densities, such as aluminium, titanium, steel and nickel-based alloys. These materials are commonly used in the oil and gas, ship, automotive and aerospace industries and require non-destructive inspection via XCT for the purpose of quality assurance. The errors introduced into XCT data by non-linear polychromatic X-ray attenuation are called beam hardening artefacts; these artefacts degrade the quality of the XCT data and can influence quantitative analysis such as porosity measurements [1] and dimensional measurements [2]. These beam hardening artefacts can be minimised or removed through algorithmic correction of the acquired data [3]. To numerically calculate a beam hardening correction function that is based on physical modelling of the XCT system, the relative intensity of each X-ray energy in the X-ray spectrum must be known, alongside the quantum detection efficiency (QDE) of the X-ray detector and the material composition of the scanned part. A number of beam hardening correction algorithms exist that do not model the underlying physics of an XCT system; such methods seek to find a correction function that best linearises the X-ray attenuation values, and these methods are typically iterative in nature [4,5] or require an additional XCT scan [6]. In this work, a method for estimating the product of the X-ray spectrum and the detector’s QDE from attenuation measurements is offered and its application for beam hardening correction is demonstrated.

### 1.1. X-ray Spectrum and Quantum Detection Efficiency

A simulated polychromatic X-ray spectrum is shown in Figure 1; it is simulated to have been generated by an X-ray source with a tungsten target and an acceleration voltage of 100 kV using the model from [7]. The X-ray spectrum is composed of discrete characteristic X-rays and broadband bremsstrahlung X-rays. Characteristic X-rays are emitted when an electron of sufficient energy collides with and ejects an inner-shell electron of an atom, and an outer-shell electron will fill the vacancy and emit X-rays that are characteristic to the given element. Bremsstrahlung X-rays are generated by the deceleration of electrons that occurs when they are deflected by an atomic nucleus. This work is concerned with estimating the bremsstrahlung distribution as it is predominantly this portion of the X-ray spectrum that causes beam hardening.

**Figure 2:**
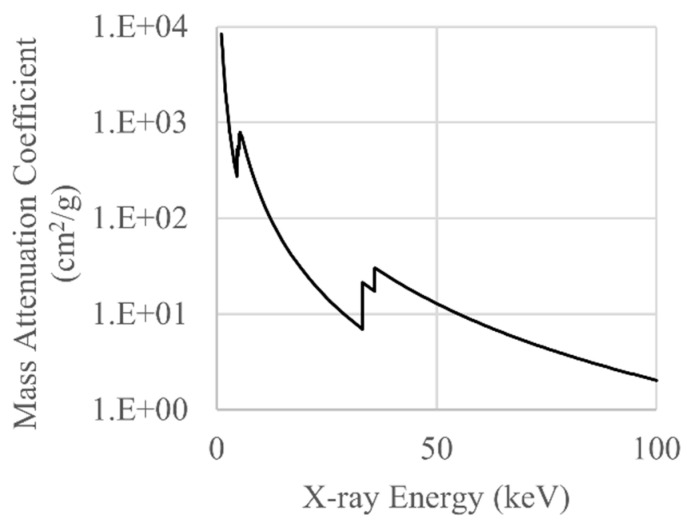
The mass attenuation coefficient for CsI plotted for the same energy range as the X-ray spectrum shown in Figure 1. Data downloaded from [8].

**Figure 3:**
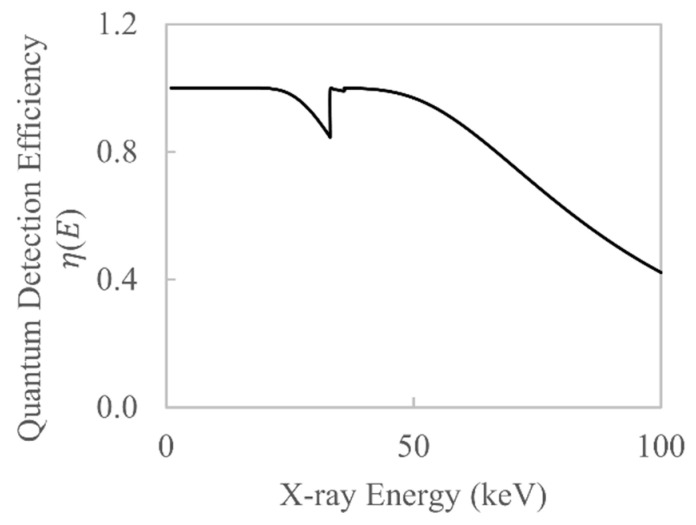
Quantum detection efficiency for a 600-µm-thick CsI scintillator calculated using Equation (1).

Indirect flat-panel detectors use a scintillator to convert X-ray photons to optical photons and then a photodiode array to convert optical photons to an electric charge for subsequent digitisation. Scintillation occurs when the scintillator absorbs an X-ray photon and sufficient energy is transferred, and the probability of absorption depends on the linear attenuation coefficient of the scintillator material. Caesium iodide (CsI) is a popular scintillator used for industrial XCT; the mass attenuation coefficient for CsI is plotted in Figure 2 for the same energy range as the spectrum shown in Figure 1. Clearly, the probability of interaction is much higher for lower X-ray energies than it is for higher X-ray energies. In addition to the energy-dependent X-ray absorption of the scintillator, the photodiode array will preferentially detect optical photons of certain wavelengths. The combined influence of the scintillator material and the photodiode array on the energy dependence of X-ray detection is termed the quantum detection efficiency (QDE) and is often modelled simply as

(1) $$\eta \left(E\right)=1-\exp\left[-\mu_{d}\left(E\right)t_{d}\right],$$

where $\mu_{d}$ is the linear attenuation coefficient of the scintillator, $t_{d}$ is the scintillator thickness and E is the X-ray photon energy. Note that this simple model does not take into account the quantum efficiency of the photodiode array. Using this model, η(E) is plotted in Figure 3 using the mass attenuation coefficients from Figure 2, assuming the density of CsI is 4.51 g/cm^3^ and the scintillator thickness is 600 µm.

### 1.2. Beam Hardening

As an X-ray beam passes through a material object, the low-energy (soft) X-rays of the spectrum are more easily absorbed then the higher-energy (hard) X-rays. As the soft X-rays are removed from the X-ray beam, the mean energy of the X-ray spectrum increases (the beam becomes harder); thus the X-ray beam becomes more penetrating. This process is called beam hardening. Ultimately, beam hardening results in the relationship between X-ray attenuation and material thickness becoming non-linear, which causes artefacts in the reconstructed XCT data because this relationship is assumed to be linear by the filtered backprojection reconstruction algorithm [9]. Beam hardening artefacts are usually corrected by estimating and applying a function that converts the non-linear attenuation to linear attenuation, which is termed linearisation [10]. Linearisation functions can be experimentally determined by making attenuation measurements of a step wedge made from the same material as the scanned object [11]. Clearly, this is not practical if an XCT user has to scan many objects, each made of a different material; in this case, a step wedge of each material is required to derive the corresponding beam hardening correction, which is expensive and time-consuming. A more general solution is to derive the linearisation function via simulation, which requires an estimate of the X-ray spectrum and the QDE of the X-ray detector [12], this being the purpose of the present work.

### 1.3. Estimating the Product of the X-ray Spectrum and QDE

An X-ray spectrum can be measured using a spectrometer [13], but these instruments are expensive and difficult to set up. Alternatively, one could simulate the X-ray spectrum using a deterministic model [14,15], but these simulations are often based on simplifying assumptions and do not accommodate all industrial X-ray source types. Monte Carlo simulations are another option [16,17], but these are typically computationally expensive and require complex modelling of the underlying X-ray–matter interactions. Another option is to estimate the product of the X-ray spectrum and the QDE from attenuation measurements [18,19,20]; this approach is simple to implement and is sufficiently accurate to derive a beam hardening correction function.

The core method for estimating the product of an X-ray spectrum and the QDE from attenuation measurements is now described. Polychromatic X-ray attenuation is written as

(2) $$-\ln\left(\frac{I}{I_{0}}\right)=-\ln\left(\int_{E_{min}}^{E_{max}}W\left(E\right)\exp\left[-\mu \left(E\right)t\right]dE\right),$$

where $I_{0}$ is the intensity of the X-ray beam that is incident on an object of thickness t and having linear attenuation coefficients μ(E), which are a function of X-ray energy E. The term I is the intensity of the X-ray beam that passes through the object without undergoing an absorption or scattering interaction. W(E) is the product of the X-ray spectrum S(E) and the QDE of the X-ray detector η(E):

(3) W(E)=S(E)η(E),

where

(4) $$\int_{E_{min}}^{E_{max}}W\left(E\right)dE=1.$$

Equation (4) is used to normalise W(E) such that each photon is weighted with respect to the total spectral characteristics of the XCT system. Knowledge of the individual terms S(E) and η(E) is not required for beam hardening correction; only their product is required. Knowledge of just the X-ray spectrum may be of interest in medical CT applications in order to assess the radiation dose received by a patient.

Equation (2) may be discretised and rewritten in matrix notation:

(5) $$-\ln\left(\frac{I}{I_{0}}\right)=-\ln\left(\left[\begin{array}{ccc} \exp\left(-\mu_{1}t\right) & \ldots & \exp\left(-\mu_{m}t\right) \end{array}\right]\left[\begin{array}{c} W_{1}\\ \vdots \\ W_{m} \end{array}\right]\right).$$

In Equation (5), all the terms are known, other than the column vector W. The ratio $-\ln\left(I/I_{0}\right)$ is the measured X-ray attenuation for a given material thickness, μ is a row vector of energy and material-dependent linear attenuation coefficients that can be looked up from an online database and t is a known material thickness through which X-ray attenuation is measured. By conducting multiple X-ray attenuation measurements through an object of a known material and a known thickness, a system of linear equations Ax=b is formed that can be solved to estimate the unknown W:

(6) $$-\ln\left[\begin{array}{c} \frac{I_{t_{1}}}{I_{0}}\\ \vdots \\ \frac{I_{t_{m}}}{I_{0}} \end{array}\right]=-\ln\left(\left[\begin{array}{ccc} \exp\left(-\mu_{1}t_{1}\right) & \ldots & \exp\left(-\mu_{n}t_{1}\right)\\ \vdots & \ddots & \vdots \\ \exp\left(-\mu_{1}t_{m}\right) & \ldots & \exp\left(-\mu_{n}t_{m}\right) \end{array}\right]\left[\begin{array}{c} W_{1}\\ \vdots \\ W_{n} \end{array}\right]\right).$$

Unfortunately, as elegant as this approach seems, the A matrix is typically ill-conditioned in that it has a high condition number and is close to singular and must therefore be solved iteratively; a thorough mathematical discussion on the topic is given by Sidky et al. [18]. The expectation-maximisation (EM) algorithm has been shown to be a suitable iterative method to solve for W, as evaluated by Sidky et al. [18] and Zhang et al. [19]. The issue with estimating W in this manner is that the intensity of each X-ray energy is an unknown, so for an X-ray spectrum with a maximum photon energy of 100 keV and energy bins of 1 keV, there are 100 unknowns to be estimated. The number of unknowns becomes much larger for industrial XCT, where X-ray energies of 450 keV and higher are not uncommon [21].

The authors note that the shape of W(E) is relatively simple if both the characteristic X-rays of the source and the absorption discontinuities of the detector are ignored. This is a reasonable simplification to make because previous work has shown that beam hardening is dominated by the attenuation of bremsstrahlung X-rays [22] and the QDE curve remains rather smooth (see Figure 3). Based on this simplification, we propose the following hypothesis: the function W(E) can be approximated with a simple analytical function. If this hypothesis holds true then the number of unknowns that require estimating can be significantly reduced, from hundreds to just a few, which, in turn, simplifies the estimation process. In this work, a rational Bézier curve is chosen to approximate W(E).

### 1.4. Rational Bézier Curves

A Bézier curve is a parametric curve that uses Bernstein polynomials as a basis. Bézier curves are chosen to approximate the bremsstrahlung distribution of the X-ray spectrum as they are simple to implement and the curve can be controlled globally by editing control points and weights [23]. Furthermore, a Bézier curve passes through the two end points, which in this case represent the minimum and maximum energies of the spectrum; it is necessary to have control over these two points. A rational Bézier curve B(t) is written as

(7) $$B\left(t\right)=\frac{\sum_{k=0}^{p}\left(\begin{array}{c} p\\ k \end{array}\right)t^{k}{\left(1-t\right)}^{p-k}a_{k}w_{k}}{\sum_{k=0}^{p}\left(\begin{array}{c} p\\ k \end{array}\right)t^{k}{\left(1-t\right)}^{p-k}w_{k}}$$

where t increases from 0 to 1 in constant steps; the step size is user-defined; p is the degree of the curve, $a_{k}=\left(x_{k},y_{k}\right)$, where x,y are the coordinates of the control points; and the terms $w_{k}$ are scalars that weight each coordinate control point.

## 2. Proposed Method

The objective of this work is to estimate W(E) by measuring X-ray attenuation through different thicknesses of material objects with known linear attenuation coefficients.

Let r be a column vector of X-ray attenuation measurements that have been measured through an object with linear attenuation coefficients μ and thicknesses $t_{1}$ to $t_{m}$. Let $\bar{r}$ be a column vector of X-ray attenuation values that are calculated using a discretised estimate of W(E), denoted by $\bar{W}$, values of μ from a reference database and reference measurements of $t_{1}$ to $t_{m}$, that is,

(8) $$\left[\begin{array}{c} {\bar{r}}_{1}\\ \vdots \\ {\bar{r}}_{m} \end{array}\right]=-\ln\left(\left[\begin{array}{ccc} \exp\left(-\mu_{1}t_{1}\right) & \ldots & \exp\left(-\mu_{n}t_{1}\right)\\ \vdots & \ddots & \vdots \\ \exp\left(-\mu_{1}t_{m}\right) & \ldots & \exp\left(-\mu_{n}t_{m}\right) \end{array}\right]\left[\begin{array}{c} {\bar{W}}_{1}\\ \vdots \\ {\bar{W}}_{n} \end{array}\right]\right).$$

Rather than solving for $\bar{W}$ directly, we propose to approximate $\bar{W}$ with a rational Bézier curve that has p+1 control points; by doing this, we greatly reduce the number of unknowns that need to be solved, hence reducing the computational complexity of the problem at hand.

The objective function to be minimised is

(9) $$\overset{m}{\underset{i=1}{\sum }}\left|r_{i}-{\bar{r}}_{i}\right|.$$

An optimisation algorithm is used to find the control points of the rational Bézier curve that minimises the objective function.

## 3. Materials and Methods

We first verify the proposed method using simulated X-ray attenuation values, whereby W(E) is known such that the accuracy of the method can be determined (Section 3.1). Following simulation-based verification, the method is applied to experimentally measured X-ray attenuation values; in this case, W(E) is unknown (Section 3.2). The experimentally estimated W(E) is finally used to derive a beam hardening correction, which is applied to a scan of an aluminium workpiece (Section 3.3).

### 3.1. Simulation-Based Verification and Benchmarking

The proposed method is first verified using simulated data, whereby a known W is used to calculate X-ray attenuation r for different materials of different thicknesses. The proposed method is used to estimate $\bar{W}$ such that the accuracy of the estimated method can be evaluated. 

X-ray attenuation is calculated using Equation (6). The W used in the calculation is shown in Figure 9; it is the product of the X-ray spectrum shown in Figure 1 and the QDE shown in Figure 3. The X-ray spectrum is simulated using Boone and Seibert’s method [7] for a tungsten anode, with $E_{min}=$ 10 keV, $E_{max}=$ 100 keV and n= 30, i.e., energy bins of 3 keV. The QDE is simulated as per the description in Section 1.1. Linear attenuation coefficients of aluminium and steel are used in the calculation and are obtained from the NIST XCOM online database [8]; these represent μ. Multiple materials are used in the simulation as this leads to a better estimate of $\bar{W}$, since the different materials attenuate each X-ray energy differently; the greater the difference in the linear attenuation coefficients, the better the estimate of $\bar{W}$, as shown by Sidky et al. [18]. The material thicknesses used in the calculation range from 0.01 to 0.1 cm in steps of 0.01 cm; these form t.

**Figure 4:**
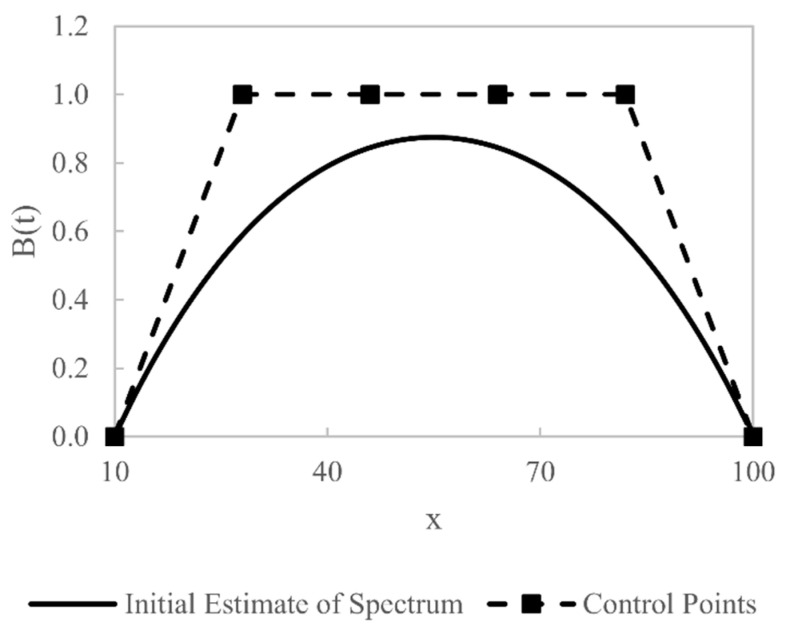
Plot of the initial estimate of W(E) using a Bézier curve and its control points.

A rational Bézier curve with 6 control points is used (p= 5). The influence of the number of control points is investigated by changing this parameter from 4 to 15; it is found that using more than 6 control points does not lead to significant improvements in the accuracy of W(E); hence we choose to use 6 control points in this work. The 6 control points are evenly distributed between $x=E_{min}$ and $x=E_{max}$. All 6 of the x coordinates are fixed, as are the first and last y coordinates with $y_{1}=0$ and $y_{6}=0$; the 4 central y coordinates are, however, free to be varied by the optimisation algorithm, alongside their respective weights, thus giving 8 optimisation variables. The initial values of the free y coordinate variables of the rational Bézier curve $y_{k}$ are all set to 1, as are the initial values of the weights, $w_{k}$. The initial shape of $\bar{W}$ with these parameter values is shown in Figure 4.

The minimisation of the objective function is performed in MATLAB R2016a (MathWorks, Natick, MA, USA) using the sequential quadratic programming algorithm with a positivity constraint on the optimisation variables. This constraint is to ensure that $\bar{W}$ does not have any negative values, since X-ray intensity cannot be negative.

The number of energy bins used in this work was chosen by means of a convergence study; energy bin widths of 2, 3, 5 and 10 keV were used to simulate polychromatic attenuation through 0.1 to 1 cm of aluminium in steps of 0.1 cm. It was found that energy bin widths of 2 and 3 keV led to attenuation errors of less than 1% when compared to a bin width of 1 keV, whist energy bin widths of 5 and 10 keV led to attenuation errors of up to 2% and 5% respectively.

To benchmark the proposed Bézier curve-based algorithm, a comparison is made to the EM algorithm as per the work of Zhang et al. [19]; see Equation (10). The EM algorithm is used to estimate all 30 energy bins of $\bar{W}$ based on the same set of simulated X-ray transmission values, $I_{i}/I_{0}$, described above. The EM algorithm is started using the same initial estimate of $\bar{W}$ as the Bézier-curve-based algorithm.

(10) $$\bar{W}\prime_{j}=\frac{{\bar{W}}_{j}}{\sum_{i=1}^{m}A_{ij}}\overset{m}{\underset{i=1}{\sum }}\frac{A_{ij}r_{i}}{\sum_{k=1}^{n}A_{ik}{\bar{W}}_{k}}$$

(11) $$A_{ij}=\exp\left(-\mu_{j}t_{i}\right)$$

(12) $$r_{i}=I_{i}/I_{0}$$

### 3.2. Experimental

**Figure 5:**
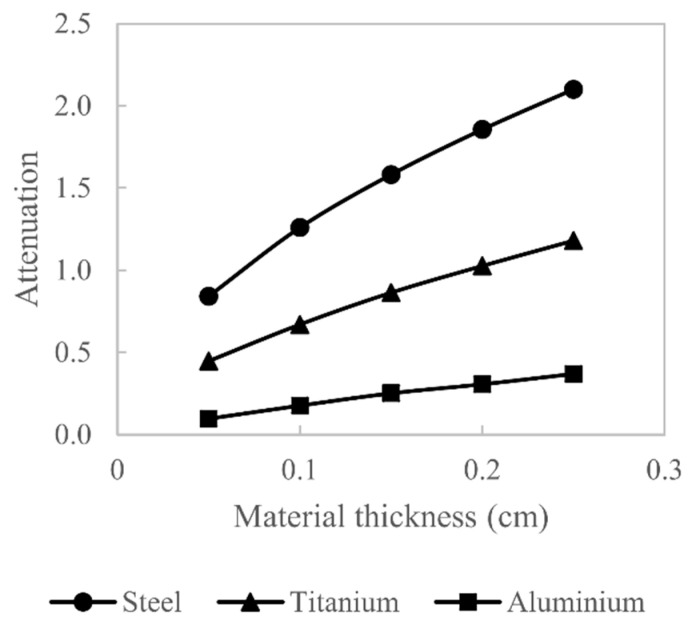
Polychromatic attenuation measurements for steel, titanium and aluminium.

Polychromatic attenuation is measured for three materials: aluminium (99% pure), titanium (99% pure) and AISI 316 stainless steel. Attenuation is measured for material thicknesses ranging from 0.05 to 0.25 cm in steps of 0.05 cm. The attenuation measurements are performed using an XYLON Y.FOX cone-beam XCT system with a 160 kV tungsten transmission target X-ray source and a Varian Paxscan scintillator-based X-ray detector with a pixel size of 0.127 mm. The attenuation measurements are made with a source-to-object distance of 286 mm and an object-to-detector distance of 412 mm. The X-ray source voltage is 140 kV, the current is 9 µA and the exposure time is 1 s. To minimise scatter contaminating the attenuation measurements and thus introducing an additional non-linearity, the X-ray source is collimated to a fan beam. The attenuation measurements are plotted in Figure 5.

The Bézier curve is defined with $E_{min}=$ 14 keV, $E_{max}=$140 keV and energy bins of 2 keV, giving n= 63; the control points are evenly spaced between $E_{min}$ and $E_{max}$. The initial conditions are as described in the previous section. The Bézier curve is evaluated using only the steel and aluminium attenuation measurements, and the estimated W(E) is then used to calculate the X-ray attenuation for titanium in order to test the accuracy of the estimate.

### 3.3. Beam Hardening Correction

**Figure 6:**
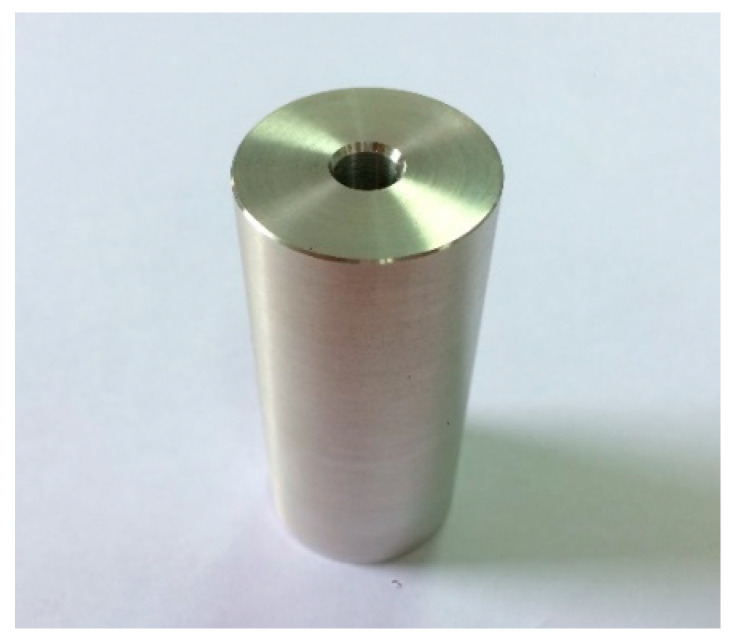
Photograph of the aluminium part used as a case study for the beam hardening correction; the nominal outer diameter is 25 mm, and the nominal inner diameter is 6 mm.

The estimated W(E) is used to derive a beam hardening correction for a scan of an industrial workpiece. The workpiece considered is an aluminium (6082-T6) cylinder turned on a lathe; the nominal inner and outer diameters are 6 and 25 mm, respectively (see Figure 6). This object is chosen because the homogenous workpiece cross section will induce a strong cupping artefact, whilst the central hole will be reconstructed with a lower contrast compared to the external background as a consequence of beam hardening. With a beam hardening correction applied, the cupping artefact should be removed and the central hole should be reconstructed with the same contrast as the external background.

The workpiece is scanned using the X-ray source settings and magnification described in the previous section. The number of projections acquired is 720, and each projection is averaged 2 times. The data is reconstructed using an in-house implementation of the filtered backprojection algorithm [9] into 512 × 512 CT images as 32-bit floating point numbers; the reconstruction filter is the Hann filter, and a cubic spline interpolation scheme is used during backprojection.

**Figure 7:**
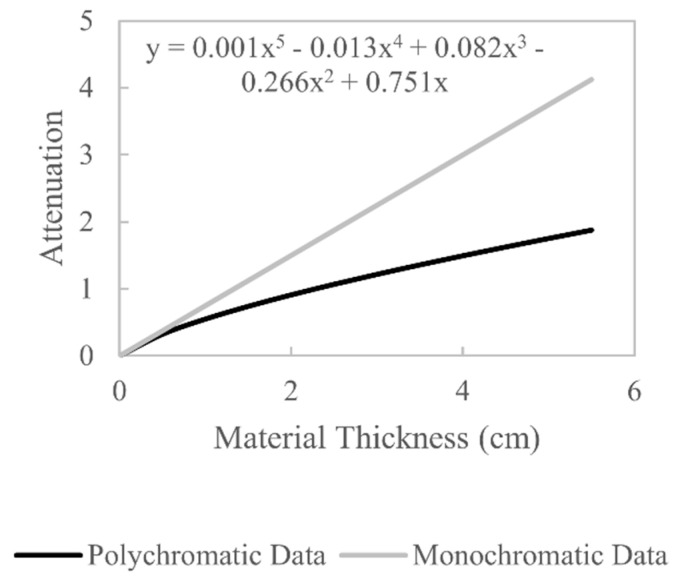
Polychromatic and monochromatic attenuation values for increasing material thicknesses. The polychromatic attenuation values are simulated using the estimated W(E). A polynomial function is fitted to the polychromatic data, and the first-order coefficient is the gradient of the monochromatic line.

**Figure 8:**
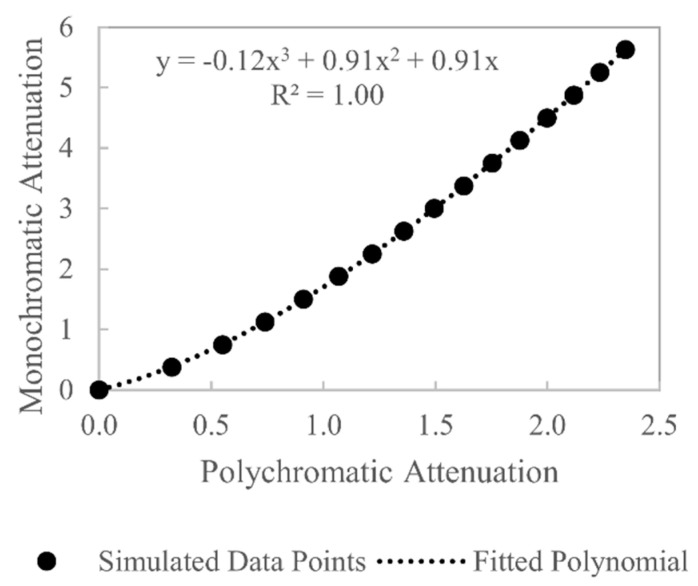
Beam hardening correction curve derived from the polychromatic and monochromatic attenuation curves in Figure 7. The correction curve is approximated using a third-order polynomial, which is a good fit, as shown by the coefficient of determination.

A linearisation beam hardening correction function is derived by plotting polychromatic X-ray attenuation against the desired monochromatic X-ray attenuation, and the resulting curve is then approximated with a polynomial function. The first step in deriving this correction function is to evaluate the relationship between polychromatic attenuation and material thickness using Equation (2). The term W(E) is estimated using the proposed spectrum estimation method, μ(E) is obtained from the NIST XCOM online database [8] (for 6082-T6: 95.35% Al, 0.25% Cr, 0.1% Cu, 0.5% Fe, 1.2% Mg, 1% Mn, 1.3% Si, 0.1% Ti, 0.2% Zn) and t is a vector of material thicknesses from 0 to the maximum path length through the object. Evaluating Equation (2) gives the polychromatic curve in Figure 7. The second step in deriving the beam hardening correction is to evaluate the relationship between monochromatic attenuation and material thickness, which is written as

(13) $$-\ln\left(\frac{I}{I_{0}}\right)=\mu \left(E\right)t$$

which is of the form y=mx, a straight line that passes through the origin. Thus, a polynomial is fitted to the polychromatic curve, and the coefficient of the first-order term is set as the linear attenuation coefficient μ(E) of the monochromatic attenuation [24]. The value of μ(E) is chosen in this manner as it represents the linear attenuation coefficient that would arise if the energy distribution of the polychromatic X-ray spectrum were unchanged as it passed through the workpiece. Evaluating Equation (13) gives the monochromatic line in Figure 7. With an estimate of the polychromatic and monochromatic attenuation values for the same material thicknesses, the desired beam hardening correction curve can be estimated by plotting polychromatic values (x-values) against monochromatic values (y-values), which is shown in Figure 8. Fitting a polynomial function to this curve allows polychromatic x-values to be converted to monochromatic y-values; thus beam hardening can be corrected by applying this polynomial function to all the projection data prior to reconstruction.

## 4. Results

### 4.1. Simulation-Based Verification

**Figure 9:**
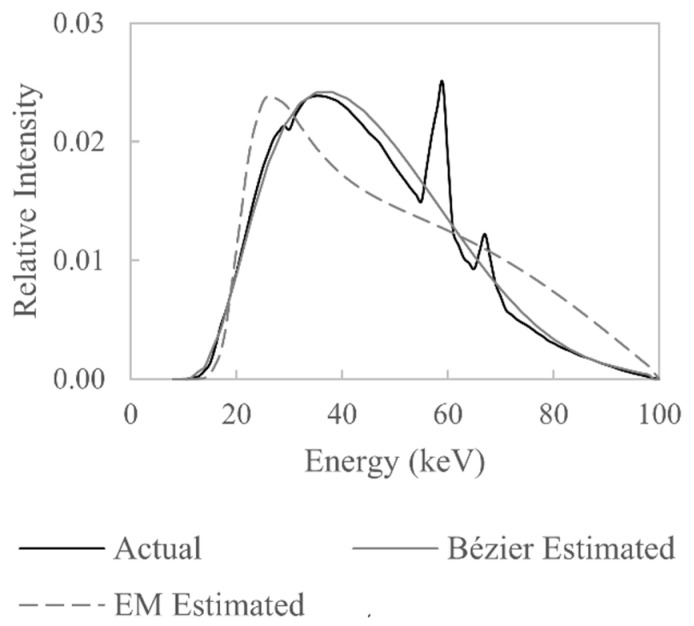
Comparison between the actual W(E) used to simulate a set of attenuation values and the W(E) estimated from the simulated attenuation values.

The estimates of W(E) based on the Bézier-curve-fitting algorithm and the EM algorithm are shown, alongside the actual W(E), in Figure 9. Clearly there is some difference between the Bézier estimated curve and the actual W(E); most notably, the characteristic X-rays are not present in the estimate and the peak bremsstrahlung energy is slightly displaced. However, given that only 8 optimisation parameters were used, the estimate is reasonable as the general shape of W(E) has been resolved. The optimisation algorithm used for the Bézier curve estimate converges after approximately 20 iterations.

The EM algorithm yields a result quite different from the actual W(E). The characteristic X-rays of the EM estimate are not recovered, and the general shape of the bremsstrahlung distribution is distorted. This is perhaps due to the use of only two materials in the formation of the system of linear equations from which W(E) is estimated; the use of only a few materials has been shown by Sidky et al. to lead to an ill-conditioned system of equations [18]. The result shown for the EM algorithm is for 10,000 iterations, and the shape of the estimated W(E) does not change significantly after 800 iterations.

**Figure 10:**
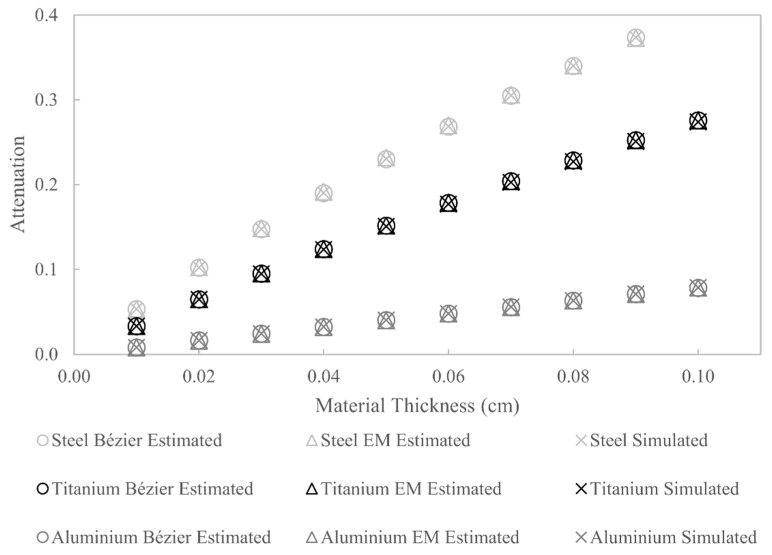
Comparison between simulated and estimated X-ray attenuation for aluminium, titanium and steel.

What is more important in this work is that the estimated W(E) can be used to accurately simulate polychromatic attenuation. A comparison between polychromatic attenuation simulated using the actual W(E), Bézier-curve-estimated W(E) and EM-estimated W(E) is shown in Figure 10. The results show that the Bézier curve estimate of W(E) can, indeed, be used to accurately simulate polychromatic X-ray attenuation, not only for the materials used in the optimisation (aluminium and steel) but also for materials not used in the optimisation (titanium). The percentage difference between attenuation values calculated using the actual W(E) and the Bézier curve estimate of W(E) does not exceed 0.24% for all three materials. For the attenuation values calculated using the EM estimate of W(E), the largest percentage difference is 1.08%; this shows that even though the estimated W(E) does not resemble the actual W(E), the estimated W(E) can still be used to accurately simulate polychromatic attenuation.

The simulation-based verification and benchmarking of the proposed method shows that the Bézier-curve-based approach leads to a more accurate representation of the shape of W(E) than the EM approach and also leads to more accurate polychromatic attenuation simulations. This is perhaps due the fact that the Bézier-curve-based approach does not seek to solve a system of linear equations directly, but rather seeks to find a parametric curve that minimises the error in the simulation of attenuation values. This statement is speculative and will require numerical investigation in future work.

### 4.2. Experimental

**Figure 11:**
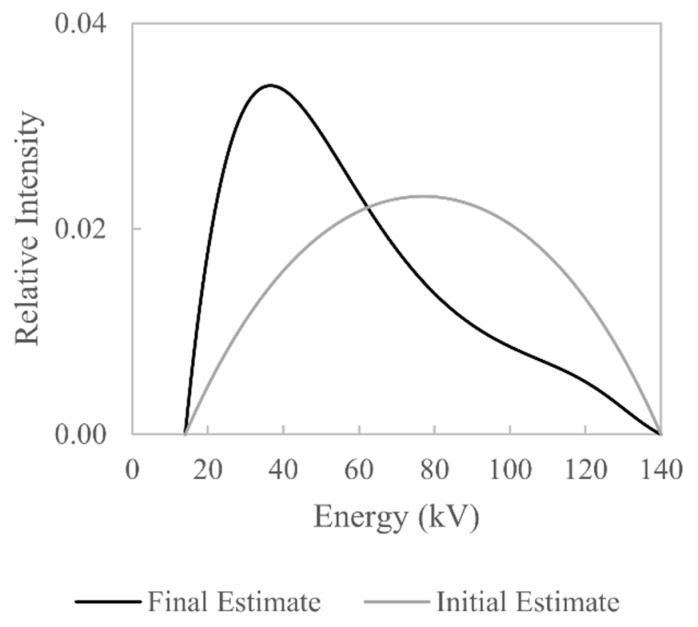
Comparison between initial and final estimated X-ray spectra based on measured attenuation values.

The estimate of W(E) based on the X-ray attenuation measurements (aluminium and steel) is shown in Figure 11, alongside the initial estimate. The general shape of W(E) is what would be expected: a higher relative intensity for the low-energy X-rays and a lower relative intensity for higher-energy X-rays.

**Figure 12:**
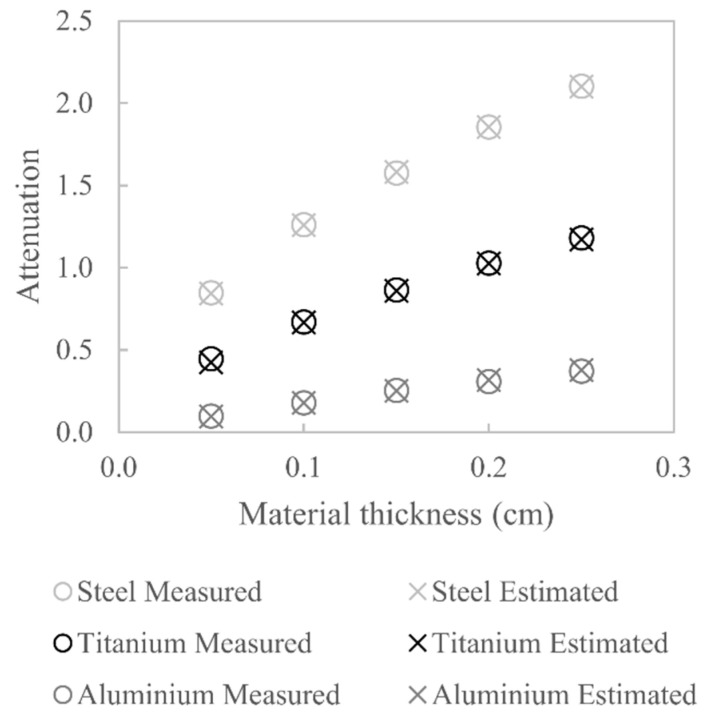
Comparison between measured and estimated X-ray attenuation for aluminium, titanium and steel.

Figure 12 shows the measured attenuation values alongside the estimated attenuation values for the three different materials considered; clearly, good agreement between the measured and estimated values is seen. The titanium attenuation measurements were not used in the optimisation; nevertheless, there is good agreement between measured and estimated attenuation values, which indicates that the estimated W(E) is close to the actual W(E). The percentage difference between the measured and estimated attenuation does not exceed 5% for all three materials.

### 4.3. Beam Hardening Correction

**Figure 13:**
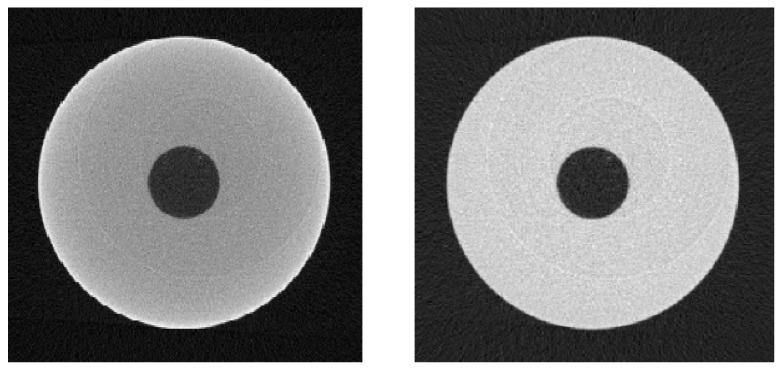
Transverse plane CT images of the aluminium workpiece. **Left**: uncorrected. **Right**: beam hardening corrected.

**Figure 14:**
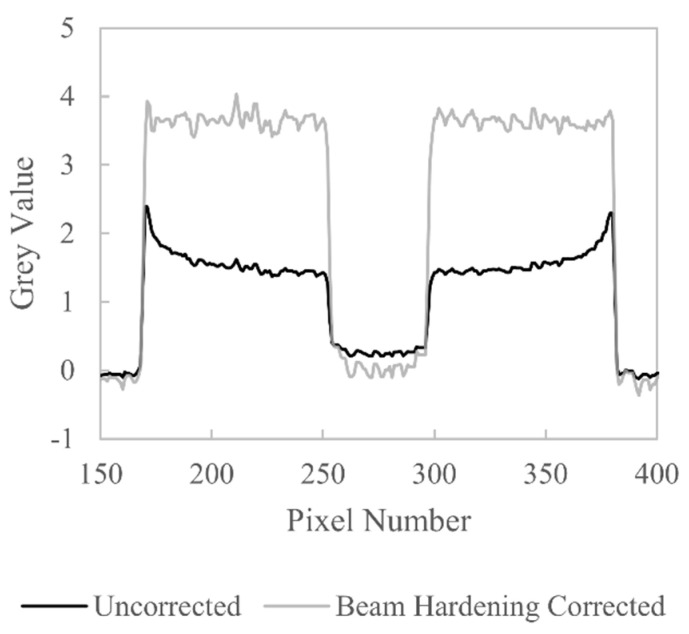
Comparison of line profiles drawn across the central row of the CT images in Figure 13.

XCT images of the aluminium workpiece are shown in Figure 13; the left XCT image is for the uncorrected data, and the right XCT image is for the beam hardening corrected data. The uncorrected data shows raised grey values at the outer edges and lower grey values towards the centre, which is seen more clearly by looking to the line profiles in Figure 14. The line profiles are plotted for the central pixel row of the CT images in Figure 13. The uncorrected line profile shows a pronounced cupping artefact and raised background grey values in the central hole compared to the external background. For the beam hardening corrected data, the grey value inhomogeneity is greatly reduced; Figure 14 shows the cupping artefact is removed and the central hole is reconstructed with grey values similar to the background grey values.

**Figure 15:**
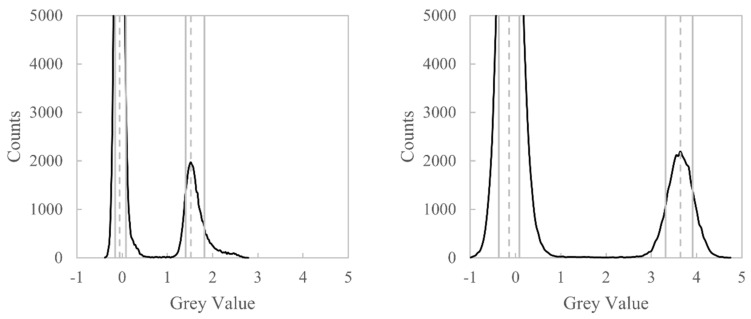
Grey value histograms of the CT images in Figure 13. The leftmost peak represents background grey values, and the rightmost peak represents the aluminium material grey values. **Left**: histogram of the uncorrected data. **Right**: histogram of the beam hardening corrected data. Grey dotted lines are the mode of each phase, whilst solid grey lines are the ±34% dispersion of each phase.

Grey value histograms evaluated from the uncorrected and beam hardening corrected data sets are shown in Figure 15. Two peaks are visible in these histograms: the leftmost peak represents background grey values, and the rightmost peak represents the aluminium grey values. The histogram for the uncorrected data shows a distortion in the aluminium material phase; this material phase should be approximately Gaussian in shape, when, in fact, it displays a positive skewness due to the raised grey values caused by beam hardening. However, after beam hardening correction, the aluminium material phase is restored to being approximately Gaussian. The histograms can be used to evaluate the grey value contrast improvement by calculating the grey value difference between the background peak and the material peak,$\;M_{b}$ and $M_{m}$, respectively. The histograms can also be used to evaluate the noise in each data set; we evaluate the ±34% dispersion (1 standard deviation) of the background and material phases, $\sigma_{b}$ and $\sigma_{m}$, respectively. The contrast-to-noise ratio (CNR) can therefore be calculated as

(14) $$\frac{M_{m}-M_{b}}{\sqrt{\sigma_{b}^{2}+\sigma_{m}^{2}}}.$$

The CNR is calculated to be 6.8 and 9.6 before and after correction, respectively, this being a 41% increase.

## 5. Discussion and Conclusions

It has been shown that the product of the X-ray spectrum and QDE can be approximated with a Bézier curve. This approximation allows the number of unknowns that require estimating to be reduced from hundreds to just a few, which simplifies the estimation process considerably. The accuracy of the estimated W(E) has been demonstrated through the ability to calculate polychromatic X-ray attenuation; the percentage difference between measured and estimated attenuation values did not exceed 5% for an arbitrary material that was not used in the W(E) estimation process. Based on these results, the estimated W(E) was used to derive a beam hardening correction function for an arbitrary material (aluminium in this case, but any material could have been chosen), and the application of this beam hardening correction to an aluminium workpiece was shown to reduce cupping artefacts and increase the CNR by 41%.

Bézier curves have been used in this work to approximate the shape of W(E); in future work, alternative parametric curves could be considered, such as piecewise cubic splines; a comparison of different curves for this purpose would be a useful contribution.

The motivation for this work was to generate beam hardening corrections without the need for material-specific step wedges. With the developed W(E) estimation method, it should be possible to generate a database of W(E) for a given XCT system, which can then be used to generate a beam hardening correction for any given material, assuming the object’s material composition is known. It may be possible to estimate W(E) at large acceleration energy intervals and interpolate between intervals to reduce the effort in estimating W(E) at smaller energy intervals, this should be considered in future work.

Based on this work, the following conclusions can be drawn:

- The product of the X-ray spectrum and quantum detection efficiency of a CT system can be approximated using a Bézier curve.
- The Bézier-curve-based estimate can be used to accurately simulate polychromatic X-ray attenuation.
- The Bézier-curve-based estimate can be used to derive a linearisation beam hardening correction, which can reduce cupping artefacts and increase the image contrast-to-noise ratio.
- Deriving beam hardening corrections via simulation is a more versatile and general solution than using material-specific step wedges.

## Data Availability

The data presented in this study are available on request from the corresponding author.
